# Superior silybin bioavailability of silybin–phosphatidylcholine complex in oily-medium soft-gel capsules versus conventional silymarin tablets in healthy volunteers*

**DOI:** 10.1186/s40360-018-0280-8

**Published:** 2019-01-11

**Authors:** Méndez-Sánchez Nahum, Dibildox-Martinez Miguel, Sosa-Noguera Jahir, Sánchez-Medal Ramón, J.Francisco Flores-Murrieta

**Affiliations:** 1 Liver Research Unit, Medica Sur Clinic & Foundation, Puente de Piedra 150, Col. Toriello Guerra, 14050 Mexico City, Mexico; 2 Italmex Pharma, 04850 Mexico City, Mexico; 3 National Institute of Respiratory Diseases “Ismael Cosío Villegas”, 14080 Mexico City, Mexico; 4 Superior School of Medicine, National Polytechnic Institute of Mexico, 14080 Mexico City, Mexico

**Keywords:** Liver fibrosis, Hepatoprotective, Silymarin, Silybin

## Abstract

**Background:**

Fibrosis is a response to chronic liver disease that results in excessive accumulation of extracellular matrix proteins and formation of scar tissue. Fibrosis represents a clinical challenge of worldwide significance. Several studies have demonstrated that many natural products and herbal medicines have activity against liver fibrosis, and extracts of milk thistle such as silymarin and silybin are the natural compounds most commonly prescribed for liver diseases. Therefore, we sought to assess and compare the pharmacokinetic properties and bioavailability of silybin–phosphatidylcholine complex in oily-medium soft-gel capsules and conventional silymarin tablets in healthy Mexican volunteers.

**Methods:**

We enrolled 23 healthy volunteers to participate in a prospective, balanced, blind, single-dose, two-way crossover study with a one-week washout period. Fasting participants received either 45 mg silybin–phosphatidylcholine complex or 70 mg silymarin to assess which formulation provided better bioavailability of silybin. Plasma was obtained and analysed for silybin concentration using a validated ultra-performance liquid chromatography–tandem mass spectroscopy method. Pharmacokinetic parameters were obtained by non-compartmental analysis and values were compared by analysis of variance for a crossover design. Ratios of maximum plasma drug concentration and area under the curve (AUC) were obtained and 90% confidence intervals were calculated.

**Results:**

The 23 healthy subjects (11 women, 12 men) who participated in the study were aged 22–31 years old (average: 28), average weight 64.8 kg, height 1.65 m and body mass index 23.5 kg/m^2^. Plasma levels of silybin were higher after the administration of silybin–phosphatidylcholine complex capsules compared with that after conventional silymarin tablets (*P* <  0.0001).

**Conclusions:**

The silybin–phosphatidylcholine complex in oily-medium soft-gel capsules seems to provide superior bioavailability. However, clinical studies must be performed to demonstrate its clinical relevance in the treatment of liver diseases.

**Trial registration:**

NCT03440164; registered on November 11, 2016.

## Background

Silymarin (SM) is a complex of one flavonoid (taxifolin) and at least seven flavonolignans, which are the most common class of substance present in milk thistle (*Silybum marianum*) extract [1]. These flavonolignans include silibinin, isosilibinin, silychristin, isosilychristin and silydianin [2], of which silybin (SIB) is the most prevalent substance with the most important biological activity. The relative abundance of SIB in SM can differ depending on the source of the botanical material, the provider and the extraction method [3]. However, SIB represents approximately 50 to 70% of the total composition of SM extract.

SM has been used for over 2000 years as a single-herb compound for treating liver diseases such as hepatitis, cirrhosis and jaundice, and as a remedy against poisoning from chemical and environmental toxins [1]. It has been demonstrated that SM protects against liver injury by means of its antioxidant, anti-inflammatory, anti-fibrotic, metabolic and cellular signalling effects [4–6]. In terms of cell signalling, SIB has been considered a chemopreventive and cancer-protective drug because of its effect on mitogenic signalling and cell-cycle regulation through induction of apoptosis and the inhibition of growth factor receptor-mediated mitogenic and cell survival signalling [7]. In vivo, SM and SIB have been used to manage alcoholic liver disease, non-alcoholic liver disease, liver fibrosis and cirrhosis [8]. Inhabitants in several European countries still use SIB to treat a range of hepatobiliary problems [9].

It is known that flavonolignans have poor and irregular bioavailability. The absorption rate of SM varies between 20 and 50% [10], while SIB has shown a straight-line concentration–response relationship over a concentration range of 0.5–100 μg/mL [11]. Many soluble derivatives of SIB have been synthesised to counteract its low solubility, bioavailability and absorption, including silybin bis-hemisuccinate, β-cyclodextrin complex, silybin-*N*-methyl-glucamine, silybin-11-*O*-phosphate and silybin-phosphatidylcholine [12–14]. In animal studies, silybin–phosphatidylcholine complex (SPC) has been shown to reduce oxidative stress, lipid peroxidation and collagen accumulation and, as a consequence, reduce liver damage [15]. Further, enzymatic synthesis of SIB β-glycosides such as silybin β-galactoside, silybin β-glucoside, silybin β-maltoside and silybin β-lactoside, have shown that they have improved SIB solubility [16]. Many studies have demonstrated in several chronic liver diseases that SIB and SM are safe and well-tolerated compounds with a limited adverse event profile [1, 5, 17]. Therefore, we sought to assess whether the bioavailability of SIB is increased by the administration of SPC in oily-medium soft-gel capsules compared with plasma levels of SIB achieved after the administration of conventional SM tablets.

## Methods

### Clinical protocol

The study began with 24 healthy volunteers. One volunteer dropped out because he took a non-authorized medication during the washout period. Twenty-three volunteers completed the clinical study. The volunteers of both sexes (12 men and 11 women) selected for the study were between 18 and 44 years old with body mass index ≥18 and ≤ 27. All subjects provided informed consent, and the Ethics Committee of Núcleo Clínico de Bioequivalencia, SA de CV (NABIO) and the Federal Commission for the Protection against Sanitary Risk (COFEPRIS) approved the clinical protocol. The study was performed in accordance with the principles of the Declaration of Helsinki (2013), General Health Law in Mexico and ICH-Good Clinical Practice (2005). All volunteers were healthy as assessed by electrocardiography, physical examination and the following laboratory tests: routine urinalysis, fasting blood glucose, total cholesterol, triglycerides, albumin, urea, creatinine, aspartate aminotransferase (AST), alanine aminotransferase (ALT), γ-glutamyl transpeptidase (γ-GT), alkaline phosphatase and total bilirubin and its fractions. All subjects were negative for human immunodeficiency virus, hepatitis B virus and hepatitis C virus (HCV). It was also verified that participants were free from significant cardiac, hepatic, renal, pulmonary, neurological, gastrointestinal and haematological diseases.

The study was conducted as a prospective, single-blind, randomized, two-period crossover balanced design with a one-week washout period between the doses. During each period, the volunteers were hospitalized 13 h before being given the drug. A xanthine-free meal was given 11 h before administering the drug. After a 10-h fasting period, they received a 45 mg dose of SPC in oily-medium soft-gel capsules or a 70 mg tablet of conventional SM. The drugs were given at 7:00 h directly into the volunteer’s mouth, followed by 250 mL of tap water. All volunteers fasted for 4 h after drug administration, when a xanthine-free standard breakfast was consumed. A standard meal was provided 8 h after dosing. No other meal was allowed during the “in-house” period. Systolic and diastolic arterial blood pressure (measured with a sphygmomanometer) and heart rate were recorded before and after drug administration.

The methodology used was similar to that previously described in other studies on bioavailability of different drugs.

### Formulations

The following test formulations were employed: SPC in oily-medium soft-gel capsules 45 mg (NeoCholal-S®, batch number 601031, expiration date 01/2018) made by Laboratorios Italmex S.A., Mexico City (Mexico) and SM tablets 70 mg (Legalon®, batch number 11149075, expiration date 05/2019) made by Laboratorios Takeda S.A., Mexico City (Mexico).

### Drug analysis

Blood samples (8 mL) from a suitable forearm vein were collected into heparin-containing tubes before drug administration (0) as well as at 0.16, 0.33, 0.50, 0.66, 0.83, 1, 1.25, 1.50, 2, 2.50, 3, 3.50, 4, 5, 6 and 8 h post dosing with each formulation. The blood samples were centrifuged at 4000 rpm for 15 min at 6 °C. Plasma was transferred to labelled tubes and stored at − 80 ± 20 °C until analysis. For drug analysis, 0.75 mL of methanol was added to a conical glass tube, followed by the plasma sample (0.5 mL). The tube was vortex-mixed for 90 s and then centrifuged at 4000 rpm for 15 min at 6 °C. The organic extracts were filtered through a 0.2-μm pore-size nylon Acrodisc. Aliquots (5 μL) were analysed by an ultra-performance liquid chromatography–tandem mass spectrometry model Acquity UPLC brand waters from Boston, USA. This model it was used to measure the concentration of SIB. The mobile phase consisted of 0.1% formic acid and methanol (20:80) at a constant flow rate (0.15 mL/min). One week after the first dose (i.e., after the washout period), the same procedure was repeated with the alternate drug.

### Calibration standards and quality control

The calibration curve was reproducible and linear based on the method used. Standard solutions were prepared from the stock solution by sequential dilutions to give eight concentrations: 1, 5, 10, 25, 50, 75, 100 and 150 ng/mL, as well as quality control (QC) plasma samples with concentrations of 3 ng/mL (low), 30 ng/mL (medium) and 120 ng/mL (high). Calibration standards were generated by spiking control human plasma with the respective standard solutions. The calibration standards and blanks were prepared in duplicate for each assay and were processed together with plasma samples and low (QCA), medium (QCB) and high (QCC) quality-control samples.

### Tolerability

Tolerability was assessed by monitoring changes from baseline in vital signs (blood pressure, heart rate, temperature and respiratory rate). Laboratory tests (haematology, biochemistry, liver function and urinalysis) were performed before dosing in each period and at the completion of the study. A clinician questioned subjects about adverse events occurring during the study or washout period, and recorded adverse events on an appropriate form.

### Pharmacokinetics and statistical analysis

Pharmacokinetic and statistical analyses were performed using the non-compartmental analysis in Phoenix WinNolin software (version 6.1; Pharsight Corporation, Mountain View, CA, USA). Maximum plasma drug concentration (C_max_), the area under the plasma concentration-time curve from time 0 to the last sampling time (AUC_0–t_), the area under the plasma concentration-time curve from time 0 to infinity (AUC_0–∞_), the time to reach C_max_ (T_max_), the half-life (t_1/2_) and the first-order terminal elimination rate constant (ke) were determined for each subject. Ke was obtained from the slope of the linear regression of the log-transformed concentration–time curve in the terminal phase. C_max_ and T_max_ were obtained directly from the curves. The areas under the SIB plasma concentration vs. time curves from 0 to 8 h (AUC_last_) were calculated by applying the linear trapezoid rule. The AUC_0–∞_ was determined by adding the value C_last_/ke to the calculated AUC_last_ (where C_last_ means the last detectable concentration). Pharmacokinetic parameters were compared using analysis of variance for a cross-over design to evaluate the bioequivalence of the test and reference formulations. Calculation of 90% confidence intervals for the ratio of test to reference treatment was conducted for C_max_ and AUC_0–∞_.

## Results

**Fig. 1:**
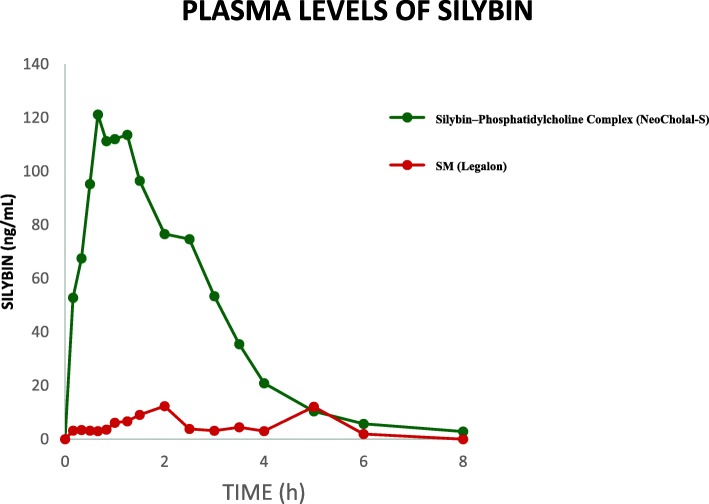
Mean silybin plasma concentrations as a function of time, obtained after single oral administrations of silybin–phosphatidylcholine complex (SPC) in oily-medium soft-gel capsules and conventional silymarin (SM) tablets (*n* = 23)

The content of SIB in the conventional tablets was 29.5 mg while that in the SPC capsules was 47.1 mg. The mean SIB plasma concentrations of the 23 volunteers after a 45 mg oral dose of SPC complex in oily-medium soft-gel capsules or 70 mg of conventional SM tablets are shown in Fig. 1. The respective mean pharmacokinetic parameters are shown in Table 1. SIB peak plasma concentrations were 207.1 mg/L for SPC and 12.6 mg/L for SM tablets. All pharmacokinetic parameters differed significantly between formulations (*P* <  0.0001). Both formulations were well tolerated with no serious adverse events observed. However, two adverse events were reported: mild epigastric pain and mild headache. The first one was related to conventional SM tablets and the second one was related to silybin-phosphatidylcholine complex.

**Table 1:** Mean pharmacokinetic parameters obtained from 23 volunteers after administration of each Silybin formulation

	SPC soft-gel capsule Mean ± SE	SM tablets Mean ± SE	*P*
T_max_ (h)	1.1 ± 0.7	1.9 ± 0.9	< 0.0001
C_max_ (ng/mL)	207.1 ± 62.4	12.6 ± 31.4	< 0.0001
AUC_0–T_ (ng/h/mL)	302.6 ± 126.6	26.4 ± 56.7	< 0.0001
AUC_0–∞_ (ng/h/mL)	308.8 ± 126.1	29.5 ± 14.1	< 0.0001
*t*_½_ (h)	1.4 ± 0.5	4.6 ± 5.8	< 0.0001

## Discussion

Many pharmacokinetic studies have shown that silybin-phosphatidylcholine improves the bioavailability of SIB in both healthy humans and in people with chronic liver disease. The present study demonstrated that SIB bioavailability is 9.6 times higher with SPC in oily-medium soft-gel capsules compared with conventional SM tablets. Several studies conducted in animals or humans have demonstrated that the complex of silybin with phosphatidylcholine has superior bioavailability over non-complexed SIB [3, 4, 18–20]. However, our results show the highest biological availability of SPC of all currently available studies.

There is still controversy about the effects of SIB on liver damage [21] because of the lack of definitive clinical data about the efficacy of SM or any of the current SIB preparations in the treatment of chronic liver disease. Some limitations of clinical studies could be related to lack of budget, small sample size, or lack of information about the type and dose of extract used and product classification [22–24]. Nevertheless, the absence of severe adverse events associated with high doses of SIB is well defined [25].

It is well known that oxidative and nitrosative stress increase the accumulation of extracellular matrix, which plays a crucial role in liver fibrosis [26]. In this context, it has been demonstrated that SPC at a dose ranging from 240 mg/d to 942 mg t.i.d. (the highest dose used in patients with liver damage) can significantly improve oxidative stress by decreasing malondialdehyde levels [3]. Therefore, this complex could be a useful antioxidant-based chemopreventive therapy to balance cellular redox. Further, some studies have evaluated the pharmacokinetics of SIB in patients with non-alcoholic fatty liver disease (NAFLD) [27, 28].

It has been reported that SM or any SIB formulation can induce a meaningful decrease in markers of chronic inflammation, metabolic parameters, degree of liver steatosis and fibrosis and an improvement in liver function tests [29, 30]. SIB has been shown to prevent mitochondrial dysfunction in animal models and to reduce liver damage in NAFLD patients [31]. In addition, similar outcomes have been reported for the use of these compounds in patients with HCV [32–37]. Consequently, SIB could be an alternative or complementary therapeutic option, particularly, when other drugs are not indicated or have failed.

## Conclusions

The presently available data demonstrates that SM or SIB formulations can be useful to treat several liver diseases. There is a need for drugs (especially for treating chronic liver disease) that can be used long-term without serious adverse events. SM and SIB formulations seem to meet this goal. This study clearly shows that SIB has superior bioavailability in healthy volunteers when administered in SPC in oily-medium soft-gel capsules compared with conventional SM tablets. However, more clinical studies must be performed to demonstrate the clinical relevance of these results for treatment of liver disease.

## Abbreviations

ALT: Alanine aminotransferase
AST: Aspartate aminotransferase
ECG: Electrocardiography
HBV: Hepatitis B virus
HCV: Hepatitis C virus
HIV: Human immunodeficiency virus
NAFLD: Non-Alcoholic Fatty Liver Disease
SIB: Silybin
SM: Silymarin
SPC: Silybin–Phosphatidylcholine Complex
γGT: γ-Glutamyl transferase
