# Correction to: Superior silybin bioavailability of silybin–phosphatidylcholine complex in oily-medium soft-gel capsules versus conventional silymarin tablets in healthy volunteers*

**DOI:** 10.1186/s40360-019-0290-1

**Published:** 2019-02-22

**Authors:** Nahum Méndez-Sánchez, Miguel Dibildox-Martinez, Jahir Sosa-Noguera, Ramón Sánchez-Medal, Francisco J. Flores-Murrieta

**Affiliations:** 1 Liver Research Unit, Medica Sur Clinic & Foundation, Puente de Piedra 150, Col. Toriello Guerra, 14050 Mexico City, Mexico; 2 Italmex Pharma, 04850 Mexico City, Mexico; 3 National Institute of Respiratory Diseases “Ismael Cosío Villegas”, 14080 Mexico City, Mexico; 4 Superior School of Medicine, National Polytechnic Institute of Mexico, 14080 Mexico City, Mexico

**Correction to: BMC Pharmacol Toxicol (2019) 20:5**

**https://doi.org/10.1186/s40360-018-0280-8**

Following publication of the original article [1], the author reported their given name have been erroneously tagged as their family names.

The correct list is:

Given name Nahum Family name Méndez-Sánchez, Given name Miguel Family name Dibildox-Martinez, Given name Jahir Family name Sosa-Noguera Jahir, Given name Ramón Family name Sánchez-Medal, Given name Francisco J. Family name Flores-Murrieta.

It has been corrected in the original article as well.

The publisher apologizes for any inconvenience caused by this error.
