# Correction to: Superior silybin bioavailability of silybin–phosphatidylcholine complex in oily-medium soft-gel capsules versus conventional silymarin tablets in healthy volunteers*

**DOI:** 10.1186/s40360-021-00500-2

**Published:** 2021-06-25

**Authors:** Nahum Méndez-Sánchez, Miguel Dibildox-Martinez, Jahir Sosa-Noguera, Ramón Sánchez-Medal, Francisco J. Flores-Murrieta

**Affiliations:** 1 Liver Research Unit, Medica Sur Clinic & Foundation, Puente de Piedra 150, Col. Toriello Guerra, 14050 Mexico City, Mexico; 2 Italmex Pharma, 04850 Mexico City, Mexico; 3 National Institute of Respiratory Diseases “Ismael Cosío Villegas”, 14080 Mexico City, Mexico; 4 Superior School of Medicine, National Polytechnic Institute of Mexico, 14080 Mexico City, Mexico

**Correction to: BMC Pharmacol Toxicol 20, 5 (2019)**

**https://doi.org/10.1186/s40360-018-0280-8**

After publication of the original article [1], an error was identified in the Results section, with regard to values of Table 1.

The incorrect sentence is:

SIB peak plasma concentrations were 207.1 mg/L for SPC and 12.6 mg/L for SM tablets. All pharmacokinetic parameters differed significantly between formulations (*P* < 0.0001).

The correct sentence is:

SIB peak plasma concentrations were 207.1 ng/L for SPC and 12.6 ng/L for SM tablets. All pharmacokinetic parameters differed significantly between formulations (*P* < 0.0001).

The original article has been corrected.
